# Postoperative footdrop following total hip arthroplasty: Epidemiology, risk factors, and associated complications

**DOI:** 10.1016/j.jor.2025.05.006

**Published:** 2025-05-04

**Authors:** Ashley Knebel, Manjot Singh, Michael J. Farias, Joseph E. Nassar, Jonathan Liu, Bassel G. Diebo, Eric M. Cohen, Alan H. Daniels

**Affiliations:** a Warren Alpert Medical School, Brown University, Providence, RI, USA; b Department of Orthopedics, Brown University, Providence, RI, USA

**Keywords:** Total hip arthroplasty, Footdrop, Complications, Risk factors

## Abstract

**Background:**

Footdrop is a relatively uncommon complication following total hip arthroplasty (THA) caused by injury to the sciatic and peroneal nerve. Few studies have evaluated the risk factors or trends for the development of this challenging complication. As such, this study aims to describe the epidemiology, procedural risk factors, and related complications of postoperative footdrop following THA.

**Methods:**

Adult primary or revision THA patients were identified using the PearlDiver database. Eligible patients were filtered into cohorts based on the development of footdrop within 90 days of surgery and matched 1:1 by age, sex, and comorbidities. Epidemiological trends in footdrop were evaluated by age and year between 2010 and 2022. Patient demographics, comorbidities, procedural characteristics, hospital outcomes, and occurrence of intra- and post-operative complications were compared.

**Results:**

In total, 2282 footdrop (0.3 %) and 893,159 (99.8 %) no footdrop THA patients were included, with a mean age of 64.9 years and 56.2 % were women. The incidence of footdrop decreased from 0.3 % to 0.2 % between 2010 and 2022 and was not associated with age. Preoperatively, footdrop patients more frequently underwent revision THA (Footdrop = 12.3 % vs No footdrop = 5.7 %, *P* < 0.001) and had more medical comorbidities (*P* < 0.001). Intraoperatively, they had higher rates of hemorrhage (0.7 % vs 0.2 %, *P* = 0.034) and nerve injury (0.7 % vs 0.1 %, *P* < 0.001). Postoperatively, they frequently reported acute kidney injury, urinary tract infection, wound-related complications, hematoma, prosthetic joint infection, aseptic loosening, and revisions (all *P* < 0.004).

**Conclusion:**

Patients developing footdrop following THA were more likely to be women, undergoing revision surgery, and had higher rates of prior lumbar fusion and medical comorbidities. They reported more postoperative complications and subsequent revisions following THA. This study informs us of the risk factors footdrop and may contribute to better patient counseling and surgical planning before undergoing THA.

## 1 Introduction

Total hip arthroplasty (THA) has been shown to provide significant improvements in pain relief, mobility, and overall quality of life for patients suffering from severe hip joint damage due to arthritis, fractures, or other conditions. In over 90 % of cases, symptomatic osteoarthritis is the reason for surgery, with the incidence of hip osteoarthritis generally increasing concurrently with obesity and age.^1^ The complication rate following THA is relatively low with reported 2.6 % and 4.2 % of patients experiencing a minor or major complication, respectively.^2^ Examples of major complications include infection, dislocations, deep vein thrombosis, and permanent nerve damage.^3^, ^4^, ^5^ One particularly concerning, though rare, complication is footdrop, which can significantly impair a patient's mobility and quality of life.^6^

Footdrop following THA is relatively uncommon, with a reported incidence ranging from 0.08 to 3.7 % following a primary procedure. It is typically caused by injury or damage to the sciatic and peroneal nerve from traction, compression, or direct trauma.^7^, ^8^, ^9^ Clinically, footdrop presents as an inability to dorsiflex the foot, leading to a characteristic high-steppage gait to prevent the toes from dragging on the ground.^9^ This gait pattern can be particularly challenging in older adults that may already struggle with balance and mobility issues. Management of postoperative footdrop following THA is not commonly reported, but typically involves bracing, physical therapy, or rarely surgical decompression in instances of nerve compression.^10^^,^^11^ Long-term outcomes depend largely on the severity of the nerve injury and the timeliness and appropriateness of the management strategies employed.

Given the relatively low incidence of footdrop following THA, few studies have reported on risk factors or trends for the development of this challenging complication. Previous studies have speculated that age over 60 years, female sex, and junior grade surgeon are all potential risk factors for developing footdrop following THA.^12^, ^13^, ^14^ Given the severe impact footdrop can have on quality of life and mobility, particularly in older patients, this study aims to describe the epidemiology, procedural risk factors, and related complications of postoperative footdrop following THA.

## 2 Methods

### 2.1 Study design

The Mariner 165 dataset on PearlDiver (PearlDiver Technologies, Colorado Springs, CO) was used in this retrospective cohort study. Between January 2010 and October 2022, over 165 million U.S. patients were covered by Medicare, Medicaid, commercial insurance, government insurance, and self-pay. International Classification of Diseases, ninth (ICD-9) and Tenth Revisions (ICD-10) and Current Procedural Terminology (CPT) billing codes were used to retrieve patient records. Since no protected health information was gathered, Institutional Review Board (IRB) review was not required.

### 2.2 Study population

Adult primary, conversion, or revision hip arthroplasty patients over the age of eighteen were identified by CPT coding (CPT-27130, CPT-27132, CPT-27134, CPT-27137, CPT-27138). Patients without at least 90 days of follow-up data were removed, as were those with a history of trauma, infection, tumors, metastases, or a preoperative footdrop diagnosis. 813,490 primary THA patients were included, of which 1864 (0.22 %) developed footdrop. 31,184 conversion THA patients were included, of which 138 (0.4 %) developed footdrop. 50,767 revision THA patients were included, of which 280 (0.6 %) developed footdrop. In total, 2282 footdrop (0.3 %) and 893,159 (99.8 %) no footdrop THA patients were included, with a mean age of 64.9 years and 56.2 % were women.

### 2.3 Cohort generation

Eligible patients were divided into two cohorts based on the development of footdrop within 90 days following surgery (footdrop vs. no footdrop) using ICD coding (ICD-9-D-73679, ICD-10-D-M21371, ICD-10-D-M21372, ICD-10-D-M21379). Following that, the patients were matched 1:1 on the following factors: age, sex, cardiac arrhythmias, chronic kidney disease, cerebrovascular disease, coronary artery disease, dementia, depression, diabetes, fluid and electrolyte disorders, hypertension, chronic obstructive pulmonary disease, obesity, liver disease, renal disease, and surgery type (primary THA vs revision THA). After matching by age, sex, and comorbidities, 2148 patients remained in each cohort, with a mean age of 65.2 years and 65.0 % were women.

### 2.4 Statistical analyses

Using linear regression modeling, epidemiological trends in footdrop were assessed by year and by age groups between 2010 and 2022. Student's *t*-test and *Chi*-square analyses were used to evaluate patient demographics and comorbidities between study groups both before and after matching. Surgery type (i.e., primary THA, revision THA) and hospital outcomes (i.e., 90-day hospital costs) were identified using ICD-9, ICD-10, and CPT billing codes and similarly compared. Finally, presence of intraoperative (i.e., hemorrhage/hematoma, nerve injury) and 90-day medical (i.e., acute kidney injury, deep venous thrombosis, pulmonary embolism, urinary tract infection), surgical (i.e., wound-related, hematoma, transfusion, site-related complication, infection), joint-related (i.e., prosthetic joint infection, aseptic loosening, periprosthetic fracture), and other (i.e., revision, admission) complications were similarly identified and compared. A *P*-value of <0.05 indicated statistical significance for patient and surgical characteristics, and a *P*-value of <0.004 using Bonferroni correction indicated statistical significance for intra- and post-operative complications. All statistical analyses were carried out using the R Statistical Package v4.2.1 (R Core Team 2022, Vienna, Austria) embedded within PearlDiver.

## 3 Results

### 3.1 Patient and procedural characteristics

**Fig. 1:**
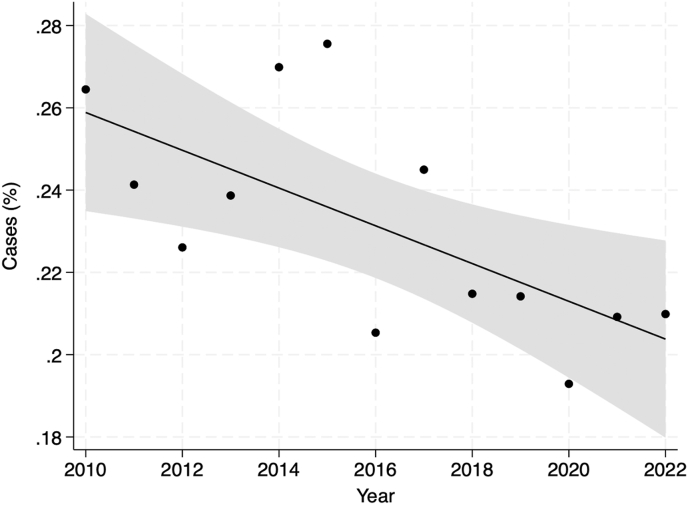
Incidence of Postoperative Footdrop by Year, *Unmatched*. Solid line represents a linear regression model fit onto the scatter plot. Grey region represents the confidence interval around the best fit line.

**Fig. 2:**
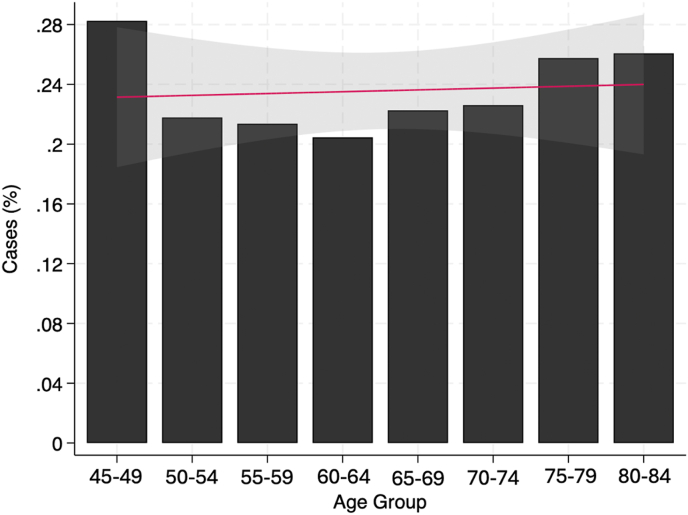
Incidence of Postoperative Footdrop by Age Group, *Unmatched*. Solid line represents a linear regression model fit onto the scatter plot. Grey region represents the confidence interval around the best fit line.

In total, 2282 footdrop (0.3 %) and 893,159 (99.8 %) no footdrop THA patients were included. Footdrop patients had a greater percent woman (65.0 % vs 56.2 %, *P* < 0.001), were more likely to have previously undergone lumbar fusion surgery (3.3 % vs 2.6 %, *P* = 0.023) were more likely to be undergoing revision THA (12.3 % vs 5.7 %, *P* < 0.001), and had higher rates of all medical comorbidities (all *P* < 0.001) than no footdrop patients. Footdrop patients also had greater 90-day total costs ($14,601 vs $9,883, *P* < 0.001) (Table 1). Between 2010 and 2022, the incidence of footdrop decreased from 0.3 % to 0.2 % (Fig. 1). The highest incidence of footdrop was 0.3 % in patients ages 45–49 years. There was no statistically significant relationship between age and the incidence of footdrop (Fig. 2).

**Table 1:** Patient demographics, comorbidities, and surgery characteristics, *Unmatched*.

Variable	Footdrop (N = 2282)	No Footdrop (N = 893,159)	*P*-value
**Demographics**			
Age (years)	65.24 (10.79)	64.94 (10.32)	0.186
Women	1484 (65.03)	501,698 (56.17)	<**0.001**
**Comorbidities (%)**			
Prior Lumbar Fusion	76 (3.33)	22,822 (2.56)	**0.023**
Cardiac Arrhythmia	866 (37.95)	290,585 (32.53)	<**0.001**
Chronic Kidney Disease	550 (24.10)	182,023 (20.38)	<**0.001**
Cerebrovascular Disease	735 (32.21)	223,484 (25.02)	<**0.001**
Coronary Artery Disease	834 (36.55)	287,205 (32.16)	<**0.001**
Dementia	201 (8.81)	59,034 (6.61)	<**0.001**
Depression	1133 (49.65)	343,233 (38.43)	<**0.001**
Diabetes	1011 (44.30)	342,102 (38.30)	<**0.001**
Fluid and Electrolyte Disorders	1095 (47.98)	325,777 (36.47)	<**0.001**
Hypertension	1917 (84.01)	717,699 (80.36)	<**0.001**
Chronic Obstructive Pulmonary Disease	839 (36.77)	281,394 (31.51)	<**0.001**
Obesity	1075 (47.11)	388,372 (43.48)	<**0.001**
Liver Disease	436 (19.11)	149,740 (16.77)	<**0.001**
Renal Disease	575 (25.20)	188,228 (21.07)	<**0.001**
**Surgery Type (%)**			
Primary Total Hip Arthroplasty	1864 (81.68)	811,626 (90.87)	<**0.001**
Conversion Total Hip Arthroplasty	138 (6.05)	31,046 (3.48)	<**0.001**
Revision Total Hip Arthroplasty	2,80 (12.27)	50,487 (5.65)	<**0.001**
**Hospital Outcomes**			
90-Day Hospital Costs	14,600.59 (22447.55)	9882.72 (14239.46)	<**0.001**

Categorical Variables are presented as count (frequency) and continuous variables are presented as mean (standard deviation).

Abbreviations: THA = Total Hip Arthroplasty.

### 3.2 Postoperative complications

Across the matched cohorts, footdrop patients had higher rates of intraoperative hemorrhage (0.7 % vs 0.2 %, *P* = 0.034), intraoperative nerve injury (0.7 % vs 0.1 %, *P* < 0.001), acute kidney injury (7.2 % vs 3.9 %, *P* < 0.001), urinary tract infection (9.5 % vs 4.9 %, p < 0.001), wound-related complications (2.0 % vs 0.9 %, *P* = 0.005), hematoma (3.4 % vs 1.1 %, *P* < 0.001), transfusion (9.7 % vs 4.4 %, *P* < 0.001), surgical site-complications (3.7 % vs 1.4 %, *P* < 0.001), infection (3.4 % vs 1.4 %, *P* < 0.001), prosthetic joint infection (4.5 % vs 1.6 %, *P* < 0.001), and aseptic loosening (5.1 % vs 1.5 %, *P* < 0.001), as well as higher rates of revisions (20.0 % vs 7.0 %, *P* < 0.001) (Table 2).

**Table 2:** Intraoperative and 90-day postoperative complications, *Matched*.

Variable	Footdrop (%) (N = 2148)	No Footdrop (%) (N = 2148)	*P*-value
**Intraoperative Complications**			
Hemorrhage/Hematoma	14 (0.65)	4 (0.19)	**0.034**
Nerve Injury	14 (0.65)	1 (0.05)	<**0.001**
**Medical Complications**			
Acute Kidney Injury	155 (7.22)	84 (3.91)	<**0.001**
Deep Venous Thrombosis	24 (1.12)	9 (0.42)	**0.014**
Pulmonary Embolism	21 (0.98)	9 (0.42)	**0.044**
Urinary Tract Infection	204 (9.50)	106 (4.93)	<**0.001**
**Surgical Complications**			
Wound Complications	42 (1.96)	19 (0.88)	**0.005**
Hematoma	72 (3.35)	24 (1.12)	<**0.001**
Transfusion	209 (9.73)	95 (4.42)	<**0.001**
Site Complications	79 (3.68)	29 (1.35)	<**0.001**
Infection	72 (3.35)	31 (1.44)	<**0.001**
**Joint-Related Complications**			
Prosthetic Joint Infection	96 (4.47)	36 (1.68)	<**0.001**
Aseptic Loosening	109 (5.07)	33 (1.54)	<**0.001**
Periprosthetic Fracture	26 (1.21)	10 (0.47)	**0.012**
**Other**			
Readmission	7 (0.33)	0 (0.00)	**0.023**
Revision	430 (20.02)	151 (7.03)	<**0.001**

Variables are presented as count (frequency).

## 4 Discussion

Despite its relatively low incidence, footdrop following THA remains a significant clinical challenge due to its impact on patient mobility and quality of life. Treatment options remain relatively limited, and many patients are left with long-term deficits and suboptimal outcomes. To our knowledge, this is the largest published series of patients with footdrop following hip arthroplasty. We identified an incidence of approximately 0.3 % following THA. Patients that developed postoperative footdrop were more likely to be women. Interestingly, age was not shown to be risk factor in this cohort, which has been reported as a risk factor in previous studies.^12^, ^13^, ^14^ Patients with footdrop also experienced higher rates of 90-day postoperative complications and revision rates in addition to greater total healthcare costs which may reflect the greater complexity of the case.

The incidence of THA has increased significantly in the last several years to approximately 544,000 cases per year as of 2024.^15^^,^^16^ We found that in the same period that THA was becoming increasingly common, the incidence of footdrop experienced a decrease from 0.3 % to 0.2 %. This decrease in the incidence of postoperative footdrop may be due to several approach- and implant-related changes aimed at reducing failure and complication rates, including increased adoption of anterior approach, larger femoral implant heads to improve stability, porous metals to improve fixation, and alternative bearings to reduce wear.^17^^,^^18^ In addition, decrease in periprosthetic fracture and dislocation may reduce nerve injury and footdrop in the acute postoperative period. We also found that revision THA had a significantly higher rate of footdrop in comparison to primary THA. Revision surgery with scar formation, increased traction, extensile exposure, and higher rates of surgical complications following revision surgery may place the peroneal and sciatic nerve at greater risk for injury and subsequent footdrop. Our study found no significant link between increased age and footdrop following THA. The highest rates of footdrop were seen in the patients ages 45–49 years, followed by patients ages 80–84 years. The high incidence of footdrop in the youngest cohort is somewhat controversial compared to prior reports of generally higher complication rates in older patients. These patients are significantly below the average reported age of 65–67 years for patients receiving THA, which could be due to extenuating circumstances such as hip dysplasia, femoral head fracture, or post-traumatic arthritis. These cases can be more technically challenging and complex, resulting in a higher incidence of footdrop in our younger patient cohort.^19^^,^^20^

Our study found that comorbidities were notably higher in patients who developed footdrop compared to those who did not. The higher comorbidity profile in our footdrop patients may reflect a complex interplay between pre-existing health conditions and the physiologic stress of surgery. Additionally, we showed that patients that developed footdrop were more likely to have previously undergone lumbar fusion surgery. This is similar to the findings of Shetty et al. who demonstrated that any history of spinal pathology, including spine surgery, with nearly 10 times greater odds of developing nerve injury following THA.^21^ This has previously been referred to as a “double” crush injury in which a patient has previously sustained a nerve injury at the lumbar spine followed by a second injury at the hip during surgery that ultimately leads to postoperative footdrop.^22^ Conditions such as diabetes can lead to nerve vulnerability and impaired healing, while cardiovascular diseases might complicate surgical outcomes and recovery. Similar to the findings of O'Brien et al. we also showed that female sex is a significant risk factor for the development of footdrop following THA.^14^ The exact mechanism by which women are at a greater risk for footdrop has not been described; however, Solhiem et al. suggests that the sciatic nerve in women may be more susceptible to damage as a result of decreased muscle mass or a change in vascular supply after giving birth.^23^

In addition, we showed that patients with footdrop experienced higher rates of medical, surgical, and joint-related complications in the postoperative period. Few studies have reported on the associated complications in patients with postoperative THA, so it is challenging to compare these findings to the literature. The higher rates of postoperative complications may be related to medical comorbidities that both predispose a patient to footdrop and our investigated outcomes. For example, higher rates of diabetes in footdrop patients may both predispose a patient to nerve injury and contribute to an elevated incidence of UTI.^24^^,^^25^ In contrast, the higher rates of hematoma or DVT may be contributory to ischemic or compressive nerve injury that ultimately leads to postoperative footdrop.^26^^,^^27^ The higher rates of both medical comorbidities and complications in footdrop patients suggests that preoperative optimization may reduce the incidence of footdrop related to non-mechanical causes while generally improving the safety of THA.

This study has several possible limitations. This was a retrospective study using a large administrative database, so it depends on accurate ICD and CPT coding. Footdrop, particularly transient cases, may not always be coded correctly which could lead to an underestimation of the true incidence. Additionally, we were unable to determine the influence of medications, such as postoperative anticoagulants, on the development of complications and ultimately the development of footdrop potentially due to inaccurate coding in the immediate postoperative period. PearlDiver does not include information about individual patients, so the results of diagnostic tests, such as nerve conduction studies, are lost. Imaging and diagnostic tests may provide more information about the mechanism and severity of the nerve damage resulting in footdrop. While several correlations with comorbidities, surgical technique, and postoperative complications were found, the true mechanism of footdrop, and its relationship to surgical case complexity and THA, could not be determined by this study.

## 5 Conclusion

In this study, we showed 0.3 % of patients who underwent THA developed footdrop. Additionally, we showed that patients with footdrop were more likely to be women, undergoing revision, and had higher rates prior lumbar fusion and of medical comorbidities prior to surgery. We also showed that patients with footdrop experienced higher rates of medical, surgical, and joint-related complications in the postoperative period. This study reports important data regarding the risk factors and associated complications of footdrop following THA. These findings may contribute to better patient counseling and surgical planning before undergoing THA.

## CRediT authorship contribution statement

**Ashley Knebel:** Conceptualization, Methodology, Writing – original draft, Writing – review & editing, Visualization, Data curation, Software, Investigation. **Manjot Singh:** Methodology, Software, Formal analysis, Investigation, Data curation, Writing – original draft, Writing – review & editing. **Michael J. Farias:** Writing – original draft. **Joseph E. Nassar:** Writing – original draft. **Jonathan Liu:** Writing – review & editing, Supervision. **Bassel G. Diebo:** Resources, Supervision. **Eric M. Cohen:** Resources, Supervision. **Alan H. Daniels:** Writing – review & editing, Resources, Supervision.

## Patient/Guardian consent

The current study did not require IRB approval nor Patient/Guardian consent because it was a retrospective cohort analysis of the PearlDiver database. PearlDiver is a national, Health Insurance Portability and Accountability Act (HIPAA)-compliant database that provides longitudinal data by utilizing administrative claims from multiple payers.

## Statements and declarations

None.

## Ethics

This manuscript has not been previously published and is not under consideration in the same or substantially similar form in any other peer-reviewed media. All authors listed have contributed sufficiently to the project to be included as authors, and all those who are qualified to be authors are listed in the author byline. BGD reports the following: receives consulting fees from Clariance and SpineVision. AHD discloses the following: receives royalties from Spineart and Stryker, consulting fees from Medtronic, research support from Alphatec, Medtronic, and Orthofix, and Fellowship support from Medtronic. The remaining authors report no conflicts of interest. No funding was received for this work.

## Funding statement

This research did not receive any specific grant from funding agencies in the public, commercial, or not-for-profit sectors.

## Conflict of interest

AK, MS, NF, MJK, JEN, and EMC have nothing to declare. BGD reports the following: receives consulting fees from Clariance and SpineVision. AHD discloses the following: receives royalties from Spineart, Stryker, and Medicrea, consulting fees from Medtronic, research support from Alphatec, Medtronic, and Orthofix, grant from Medtronic, and fellowship support from Medtronic.
